# Green LAI Mapping and Cloud Gap-Filling Using Gaussian Process Regression in Google Earth Engine

**DOI:** 10.3390/rs13030403

**Published:** 2021-01-24

**Authors:** Luca Pipia, Eatidal Amin, Santiago Belda, Matías Salinero-Delgado, Jochem Verrelst

**Affiliations:** 1 Institut Cartogràfic i Geològic de Catalunya (ICGC), Parc de Montjüic, 08038 Barcelona, Spain; 2 Image Processing Laboratory (IPL), University of Valencia, C/Catedrático José Beltrán 2, 46980 Valencia, Spain

**Keywords:** Google Earth Engine (GEE), Gaussian process regression (GPR), machine learning, Sentinel-2, gap filling, leaf area index (LAI)

## Abstract

For the last decade, Gaussian process regression (GPR) proved to be a competitive machine learning regression algorithm for Earth observation applications, with attractive unique properties such as band relevance ranking and uncertainty estimates. More recently, GPR also proved to be a proficient time series processor to fill up gaps in optical imagery, typically due to cloud cover. This makes GPR perfectly suited for large-scale spatiotemporal processing of satellite imageries into cloud-free products of biophysical variables. With the advent of the Google Earth Engine (GEE) cloud platform, new opportunities emerged to process local-to-planetary scale satellite data using advanced machine learning techniques and convert them into gap-filled vegetation properties products. However, GPR is not yet part of the GEE ecosystem. To circumvent this limitation, this work proposes a general adaptation of GPR formulation to parallel processing framework and its integration into GEE. To demonstrate the functioning and utility of the developed workflow, a GPR model predicting green leaf area index (LAI*_G_*) from Sentinel-2 imagery was imported. Although by running this GPR model into GEE any corner of the world can be mapped into LAI*_G_* at a resolution of 20 m, here we show some demonstration cases over western Europe with zoom-ins over Spain. Thanks to the computational power of GEE, the mapping takes place on-the-fly. Additionally, a GPR-based gap filling strategy based on pre-optimized kernel hyperparameters is also put forward for the generation of multi-orbit cloud-free LAI*_G_* maps with an unprecedented level of detail, and the extraction of regularly-sampled LAI*_G_* time series at a pixel level. The ability to plugin a locally-trained GPR model into the GEE framework and its instant processing opens up a new paradigm of remote sensing image processing.

## 1 Introduction

The estimation of quantitative vegetation variables is fundamental to assess the dynamic response of vegetation to changing environmental conditions [1]. Earth observation sensors in the optical domain enable the spatiotemporally-explicit retrieval of plant biophysical parameters [2]. Since the advent of optical remote sensing science, a variety of retrieval methods for vegetation attribute extraction emerged. Essentially, quantification of surface biophysical variables from spectral data always relies on a model, enabling the interpretation of spectral observations and their translation into a surface biophysical variable. Methodologically, these retrieval models can be classified into the following four categories: (1) parametric regression, e.g., spectral indices combined with a fitting function, (2) non-parametric regression, e.g., machine learning regression algorithms (MLRAs), (3) physically-based, i.e., inverting radiative transfer models (RTMs), and (4) hybrid methods. See [3,4] for a comprehensive review of these methods and mapping applications. Each of these categories has their benefits and limitations, depending on the targeted application. Hybrid methods blend the generic properties of physically-based models combined with the flexibility and computational efficiency of MLRAs. Within such a scheme, lookup tables (LUT) are generated from RTM simulations. Then, the MLRA learns the (non-linear) relationship between the pairs of reflectance and vegetation trait of interest. Hybrid methods tend to be preferred when it comes to operational processing to be applied across the globe, given their general applicability, processing speed and competitive performances. One of the major advantages of these methods is that, once a MLRA is trained, it can process an image into a vegetation product quasi-instantaneously. Current hybrid schemes for the generation of land products typically rely on neural networks (NNs) trained using a very large amount of RTM-simulated data [5]. Previous studies with NNs and LUT-based inversion even suggested LUT sizes from 8000–100,000 combinations of input variables [6,7].

In this regard, due to the fast progress in the development of machine learning techniques and their applications, for the last few years alternative MLRAs came forth as appealing alternatives over conventional NN models into hybrid retrieval strategies [8,9]. Especially, the MLRA families of decision trees and kernel-based methods proved to deliver outstanding mapping results [3,4]. These methods tend to be simpler to train, i.e., no need for such large LUTs, and for vegetation properties estimation can perform more robustly than NNs when a reduced number of training samples is available, while maintaining competitive accuracies [8,10]. From the kernel-based algorithms, noteworthy are kernel ridge regression [11], because of its simplicity and therefore fast run-time, support vector regression [12] and Gaussian process regression (GPR) [13]. GPR is particularly attractive because of carrying out statistical learning developed in a Bayesian framework. Lately, GPR became one of the main interesting kernel-based ML methods for vegetation properties retrievals: GPRs excel other ML algorithms through delivering competitive prediction accuracy [8,10,14] and insight in relevant bands [15], and above all, providing a closed form expression for the uncertainty intervals of the estimates. This distinct feature provides a valuable source of additional information, e.g., to assess the robustness of the predictions at varying spatiotemporal scales [16].

Essentially, the entire procedure of learning a GPR model only relies on an appropriate selection of the type of kernel and the hyperparameters involved in the estimation of input data covariance. Kernels contain assumptions about the function we wish to learn and define the closeness and similarity between data points. Once a kernel is selected, the unknown hyperparameters of the kernel need to be learned from the training data [17]. Trained GPRs are usually highly flexible and accurate for prediction over new inputs closer to training data points, whereas their uncertainty increases when the new inputs are further away from the available training information. With these appealing properties, apart from retrieval applications, recent studies have demonstrated the effectiveness of GPR for time series gap-filling applications [18–20].

Given the attractive properties of GPR and with ambition of progressing towards operational processing, the algorithm was recently explored as a prototype retrieval algorithm for automated processing of Sentinel-2 (S2) tiles into products of green leaf area index (LAI*_G_*) and brown LAI (LAI*_B_*) at a pixel resolution of 20 m [21]. GPR models were developed, applied to S2 imagery, and LAI maps were validated across various sites in Europe. In addition, LAI time series were processed for the selected sites; the regular occurrence of cloud cover in optical data makes that gap-filling and smoothing techniques are mandatory for obtaining meaningful phenology profiles. Among multiple gap-filling fitting functions analyzed [20,22], GPR appeared to be one of the top performing algorithms in correctly reconstructing cloud-free LAI*_G_* products [20], and at the same time providing associated uncertainties. Altogether, GPR proved to be appealing for spatiotemporal processing optical satellite data, i.e., not only for retrieving LAI from S2 data but also for gap-filling processing in order to obtain cloud-free LAI*_G_* estimates at regular intervals (e.g., each 5 days).

However, when eventually aiming for seamless S2 data processing applicable to any corner in the world, more computationally-efficient solutions have to be sought for. While GPR processes a single S2 tile reasonably fast (in the order of minutes), GPR processing becomes tedious when time series of S2 tiles must be processed, and this is especially true when covering larger regions. Moreover, preprocessing steps, such as selecting and preparing S2 tiles from the Copernicus data hub [23], adds to additional runtime and eventually becomes a bottleneck when not fully automated. Altogether, in order to achieve dynamic processing of a vast amount of S2 data, it demands for: (1) migrating towards cloud-computing platforms, and (2) integrating the GPR algorithm into the cloud-computing platforms. Hence, this leads to new challenges to overcome, but also opportunities towards interactive on-the-fly processing of vegetation properties estimation in a cloud computing environment. Specifically, for the last few years the Google Earth Engine (GEE) platform emerged as an attractive high-performance computing platform to enable cloud-based processing of petabytes of S2 data [24]. GEE provides powerful computational capability for planetary-scale data processing and allows creation and training for well-known machine learning algorithms [25]. However, despite the growing capabilities in advanced machine learning tools in GEE, the possibility to train and apply GPRs, or even just run already trained GPRs, is still lacking. This means that solutions have to be developed to enable integrating GPR into the GEE environment. All in all, the ambition to plug in GPR into the GEE platform for the generation of vegetation products from S2 satellite data in a flawless cloud-based approach brings us to the following main objectives: (1) to adapt the GPR algorithm for multispectral data so it is scalable into GEE environment; (2) to optimize and import a GPR model for LAI*_G_* estimation in GEE; (3) to process S2 multispectral data into LAI*_G_* in GEE; (4) to tackle the gap-filling of discontinuous LAI*_G_* time series by extending the GPR modeling to the time domain; and (5) exemplify the processing power of the developed framework with a few comprehensive case studies.

The remainder of the paper is structured as follows. The GPR standard theory and a simple reformulation tailored to parallelize the prediction process is described in Section 2. Section 3 outlines the data and the followed methodology for training the GPR models. Section 4 summarizes the strategy to plugin GPR into the GEE platform. Section 5 provides a demonstration case of reconstructing cloud-free composite LAI maps from Sentinel-2 acquisitions over wide areas. A discussion on the workflow’s strengths and weaknesses and future research lines is presented in Section 6, whereas conclusions are finally presented in Section 7.

## 2 Methodology

### 2.1 GPR Formulation for Vector Input

Standard GPR models are state-of-the-art statistical methods for non-parametric regression and function approximation. In recent years, we have successfully applied GPRs for the retrieval of biophysical parameters from optical imagery, see [3,14,16,26–31]. GPR models yield predictions of the phenomenon along with an estimation of their uncertainty. A general introduction to GPR can be found in [13,30]. In the following, we briefly review the standard GPR adapted to the general needs of this study. Bold font is used for variables to indicate a vector.

In general, GPR models establish a relation between the input $\text{x}\in {\mathbb{R}}^{D}$ and the output variable y∈ℝ. Assuming that *y* corresponds to noisy observations of the true underlying function *f* **(*x*)**, i.e., *y* = *f* (*x*) + *ɛ*, and that the noise *ɛ* is additive dimension-independent Gaussian distributed with zero mean and variance $\sigma_{n}^{2}$, the GPR model assumes that *f*(**x**) is a Gaussian-distributed random vector with zero-mean and covariance matrix **K**(**x**, **x**), i.e., f(x)∼𝒩(0,K). The elements *ij* of the covariance matrix are calculated by means of a kernel function *k***(*x_i_***, ***x**_j_***)**, which encodes the similarity between input vectors ***x**_i_* and ***x**_j_*. Various kernel functions, with associated kernel parameters (i.e., hyperparameters), can be employed in a GPR [13,32]: Squared Exponential (SE), Matern 3/2, Matern 5/2 and Rational Quadratic (RQ), among others. The choice of the kernel function, and consequently of its hyperparameters, is usually referred to as model selection.

In this study, we pay special attention to the most commonly employed SE covariance function (Equation (1)):

(1) $$k\left({\text{x}}_{i},{\text{x}}_{j}\right)=\sigma_{s}^{2}\text{exp}\left(-\frac{1}{2}\sum_{b=1}^{D}{\left[\frac{{\text{x}}_{i}\left(b\right)-{\text{x}}_{j}\left(b\right)}{\sigma_{b}}\right]}^{2}\right),$$

where $\sigma_{s}^{2}>0$ is the signal variance and *σ_b_* is a dedicated parameter controlling the spread of the training information along the input dimension *b*. Defining *σ* = [*σ_1_*, .., *σ_D_*], the kernel is thus parameterized by a set of hyperparameters, collectively denoted as $\theta =\left\{\sigma_{s}^{2},\sigma^{2},\sigma_{n}^{2}\right\}$. The term $\sigma_{n}^{2}$ is the variance of the additive noise affecting the input data. These free hyperparameters *θ* allow for flexible customization of the GPR for a wide variety of regression problems, having the following interpretation:

- Length-scale *σ_b_* describes the smoothness of *f* **(*x*)** dependence along the dimension *b*. Small *σ_b_* means *f* **(*x*)** changes quickly for variations of **x** along *b*; large values denote slow changes w.r.t. the *b* dimension. Alternatively, the inverse of *σ_b_* represents the relevance of band *b* in the prediction process. Intuitively, high values of *σ_b_* mean that relations largely extend along that band hence suggesting a lower informative content.
- Signal variance $\sigma_{s}^{2}$ is a scaling factor. It determines variation of *f* **(*x*)** from its mean. Small value of $\sigma_{s}^{2}$ characterize functions that stay close to their mean value, larger values allow more variation. If $\sigma_{s}^{2}$ is too large, the modeled function will be free to chase outliers.
- Noise variance $\sigma_{n}^{2}$ is formally not a part of the covariance function itself. It is used by the Gaussian process model to account for noise present in training data.

The Bayesian framework allows estimating the distribution of f_*_ at the test point x_*_ conditioned on the training data and kernel’s hyperparameters. According to the GPR formulation, *f* (x_*_) is normally distributed with mean and variance given by:

(2) $$\begin{array}{l} f\left(x_{*}\right)=k_{*}^{T}{\left(K+\sigma_{n}^{2}I_{N}\right)}^{-1}y\\ \sigma_{f}^{2}\left(x_{*}\right)=c_{*}-k_{*}^{T}{\left(K+\sigma_{n}^{2}I_{N}\right)}^{-1}k_{*} \end{array}$$

where k_*_ = [*k*(x_*_, **x**_1_), …, *k*(x_*_, ***x**_N_*)]*^T^* is an *N* × 1 vector, **y** = [*y*_1_, .., *y_N_*]*^T^* and $c_{*}=k\left(x_{*},x_{*}\right)+\sigma_{n}^{2}$.

For Gaussian Process regression with Gaussian noise, it is possible to obtain the probability of the data given the hyperparameters *p***(y|x**, **θ)** by marginalization over the function values *f* [13]. The log marginal likelihood is given by:

(3) $$\log p\left(y|x,f\right)=-\frac{1}{2}y^{T}{\left(K+\sigma_{n}^{2}I_{N}\right)}^{-1}y-\frac{1}{2}\log|K+\sigma_{n}^{2}I_{N}|-\frac{n}{2}\log2\pi$$

The first term in Equation (3) can be interpreted as a data-fit term, the second term is a complexity penalty and the last term is a normalizing constant. From the theoretical point of view, any optimization algorithm can be applied to maximize the marginal likelihood in Equation (3). Yet, its selection must take into account very carefully the complexity of the problem to be solved and the dimensionality of the training data [33]. The optimization steps mentioned in the rest of the manuscript have been carried out using the conjugate gradient method proposed in [13] and implemented in [34]. We will call this optimization procedure training the GPR [13,35].

Once the hyperparameters θ have been estimated, the prediction of *y* for a new input vector x_*_ is given along with its uncertainty by Equation (2). Note that the mean prediction is often referred to as a linear predictor, as it can be seen as a linear combination of *N* kernel functions, each one centered on a training point

(4) $$f\left(x_{*}\right)=\sum_{i=1}^{N}\alpha_{i}k\left({\text{x}}_{i},{\text{x}}_{*}\right)=k_{*}^{T}\alpha$$

where ${\left\{{\text{x}}_{i}\right\}}_{i=1}^{N}$ are the training vectors contained in the model, *k* is the Kernel function evaluating the similarity between the new input **x** and the generic training samples ***x**_i_*, *i* = 1, …, *N* and $\alpha_{i}\in \mathbb{R}$ is the element *i* of the vector $\alpha ={\left(K+\sigma_{n}^{2}I_{N}\right)}^{-1}y$.

### 2.2 GPR Formulation for Space-Spectrum (3D) Input

The training step of a GPR performs a non-linear optimization to provide at once the hyperparameters θ and the vector samples contained in the model to be compared to each new input for prediction. At this point, GPR prediction becomes a linear operation with respect to the number of model samples, as stressed by Equation (4), and a specific formulation for parallel computing strategy can be pursued. In addition, several operations which are dumbly repeated in a vector-based formulation can be pre-calculated and applied a reduced number of times at specific step of the prediction process, significantly reducing the overall computational burden of the final estimation. Being α defined once the model is trained, the problem reduces to optimize the estimation of k_*_.

Equation (1) can be expanded for the new input x_*_ and the model sample ***x**_i_* as:

(5) $$\text{k}\left({\text{x}}_{*},{\text{x}}_{i}\right)=\sigma_{s}^{2}\text{exp}\left(-\frac{1}{2}\left[{\text{x}}_{*}^{T}D{\text{x}}_{*}-2{\text{x}}_{*}^{T}D{\text{x}}_{i}+{\text{x}}_{i}^{T}D{\text{x}}_{i}\right]\right)$$

with $D=diag\left(\sigma_{1}^{-2},..,\sigma_{B}^{-2}\right)$. Grouping the N samples contained in the GPR model as the *B × N* matrix ***X* =** [**x**_1_, **x**_2_, …, ***x**_N_*], k_*_ can be obtained as:

(6) $$k_{*}=\sigma_{s}^{2}\text{exp}\left(-\frac{{\text{x}}_{*}^{T}D{\text{x}}_{*}}{2}\right)\text{exp}\left(-\frac{1}{2}\left[-2{\text{X}}^{T}D{\text{x}}_{*}+(({\text{X}}^{T}D{)}^{T}\circ \text{X}{)}^{T}J_{B,1}\right]\right)$$

(7) $$=\sigma_{s}^{2}\text{exp}\left(-\frac{{\text{x}}_{*}^{T}D{\text{x}}_{*}}{2}\right)\text{exp}\left({\text{X}}^{T}D{\text{x}}_{*}-\frac{{\left(D\text{X}\circ \text{X}\right)}^{T}J_{B,1}}{2}]\right)$$

where ○ denotes the Hadamard (o element-wise) matrix product, and ***J****_B_*_,1_ indicates the *B* × 1 unit matrix. Note that the first element within the exponent expression is a scalar term which depends just on the new input vector and model’s hyperparameters, but not on its training samples **X**; the third one is related to the information contained in the trained model, but not on the new input to be used for prediction. Accordingly, we can give one further step towards parallelization, and extend the estimation of k_*_ to a *B* × *M* matrix X_*_ = [x_*1_, .., x*_M_] containing *M* new input vectors, being *M* the number of pixels of the multispectral image to be processed at once. Defining $K_{*}=\left[k_{*}^{1},k_{*}^{2},..k_{*}^{M}\right]$, it results:

(8) $${\text{K}}_{*}=\sigma_{s}^{2}\text{exp}\left(-\frac{{\left(D{\text{X}}_{*}\circ \text{X}\right)}^{T}J_{N,1}J_{1,M}}{2}\right)\circ \text{exp}\left(-\frac{{\left(D\text{X}\circ \text{X}\right)}^{T}J_{B,1}J_{1,M}}{2}]\right)\circ \text{exp}\left({\text{X}}^{T}D{\text{X}}_{*}\right)$$

where ***J***_1,_*_M_* has been introduced to generate a replication of column vectors and achieve dimensional equality of the overall matrix expression. Note that the terms within Hadamard product in Equation (8) correspond to *N* × *M* matrices.

Finally, the expression to predict the mean value *f* (X*) provided by the trained GPR model for an input *M*-pixel image with *B* bands X* can directly be obtained by combining Equations (4) and (8) as:

(9) $$f\left(X_{*}\right)=K_{*}^{T}\left(\alpha J_{1,M}\right)=J_{M,1}\alpha^{T}K_{*}.$$

### 2.3 GPR Formulation for Space-Time (3D) Input

The formulation presented in Sections 2.1 and 2.2 can be slightly modified to deal with a different regression task. Let the maximum number of input vector dimension be known, but not the real one, i.e., the number of meaningful bands. This is the case, for instance, of any surface parameter time series from satellite optical observations taken over an area of interest with a fixed sampling rate. The nominal number of acquisitions is known once the observation period is defined, but not the number of meaningful samples for each pixel, as it depends on the presence of clouds which may cover entirely or partially the area of interest.

With respect to Equation (1), the input becomes a scalar (*D* = 1) as it corresponds to time. Then, the kernel for covariance estimation is given by

(10) $$k_{t}\left(t_{i},t_{j}\right)=\sigma_{st}^{2}\text{exp}\left(-\frac{1}{2}{\left[\frac{t_{i}-t_{j}}{\sigma_{t}}\right]}^{2}\right)$$

where *t_i_* and *t_j_* denote two generic acquisition dates of non-cloudy acquisitions from which the surface parameter of interest *P_S_* has been retrieved. Being **t** = [*t*_1_, …*t_N_t__***]***^T^* the vector of the *N_t_* sample dates for a specific pixel, the GPR estimation of *P_S_* at a new dates t_*_ is still given by Equation (2) assuming $P_{S}\left(t\right)∼𝒩\left(0,{\text{K}}_{t}\left(t,t\right)\right)$. Defining $D_{t}=\sigma_{2}^{-2}$, the covariance **K***_t_* can be calculated as

(11) $${\text{K}}_{t}=\sigma_{st}^{2}\text{exp}\left(-D_{t}\frac{\left(\text{t}{\text{J}}_{1,N_{t}}-{\text{J}}_{N_{t},1}t^{T}\right)}{2}\right),$$

and the expression to obtain the mean value prediction of *P_S_* at t_*_ becomes

(12) $$P_{S}\left(t_{*}\right)={k_{t_{*}}}^{T}{\left(K_{t}+\sigma_{nt}^{2}I_{N_{t}}\right)}^{-1}P_{S}\left(t\right)={k_{t_{*}}}^{T}\alpha_{t}$$

where **P***_S_***(t) = [***P_S_***(***t*_1_**)**, …, *P_S_***(***t_N_t__***)]***^T^* is the pixel’s parameter time series, $\sigma_{nt}^{2}$ is the variance of the additive noise of time series **P***_S_*(**t)** and $\sigma_{st}^{2}$ is the time series signal variance. It is worth mentioning that in case of cloud-free acquisition set, Equation (11) is the same for all the pixels within the scene (but not so **α***_t_*). Nonetheless, this is an unrealistic case, as the presence of clouds produces a spatial variability of the number of valid time samples, even within small areas. This pixel-wise dependence makes a parallel implementation of Equation (12) not easily achievable. A workaround for this problem, specifically designed for GEE, will be put forward in Section 4.2.

## 3 GPR Models Training

The generic formulation of GPR described in Section 2.1 can easily be employed for vegetation parameter retrieval modeling and gapfilling purposes. For the former, we can take into account the Sentinel-2 imagery and generate a model for the estimation of green LAI. For the latter, we analyze time series of green LAI over different crop regions to characterize their dynamics over time.

### 3.1 Green LAI Model

Green LAI is defined as one half the total green leaf area per unit ground surface area (hereafter referred to as ‘LAI*_G_*’) [36], and thus for crop fields, this only accounts for functioning above-ground parts of the plants that are green and photosynthetically active during a significant fraction of the growth cycle [37,38]. A key aspect about implementing an LAI*_G_* model into GEE was to ensure that a mature, well-validated GPR model was chosen. Therefore, a GPR model was selected that was developed and validated as part of a recently completed H2020 project called SENSAGRI (Sentinels Synergy for Agriculture-http://sensagri.eu/) [28]. During that 3-year project, multiple GPR models were trained and gradually improved with increasingly available empirical data of LAI*_G_* ground-based measurements collected from multiple field campaigns [21]. The corresponding reflectance information was either synthesized from hyperspectral data covering all S2 spectral bands or extracted from simultaneous S2 imagery. According to S2 band settings, only the 10 and 20 m spatial resolution bands were selected, i.e., 10 bands in total [39]. The training dataset includes diverse crop types over different locations across Europe as well as forested areas, as described in [21] and summarized in Table 1. Additionally, the training datasaet was expanded with non-vegetated areas spectra. In total, 218 data pairs samples were used to train the LAI*_G_* GPR model. The final ‘LAI*_G_*’ model was validated based on a new independent dataset collected during the summer of 2018 on agricultural sites in France, Poland and Ukraine. Detailed information about data collection and validation exercises can be found in [21].

However, this final model appeared to be too computationally demanding for running into GEE, and a lighter surrogate model had to be developed. In order to obtain an optimized training set while keeping a model size that does not compromise its portability and implementation in GEE, several active learning (AL) [40,41] algorithms were applied in the GPR training phase. AL methods are sampling reduction techniques that select the most informative samples from the original training dataset, still reaching a high retrieval model’s estimation accuracy. AL methods start with a small training set of data pairs and iteratively use selection criteria to extract samples from a larger data pool, until reaching an optimal final training database, whose size is (1) enough reduced to substantially increase computational efficiency of the final prediction model and (2) large enough to capture the original training set diversity. The selection criteria aims to extract, from the input larger data pool, those samples which would contribute the most to improve the regression model [40,41].

The training procedure and AL analysis was conducted within the Automated Radiative Transfer Models Operator (ARTMO) scientific package [34] https://artmotoolbox.com/, using the MLRA toolbox, which contains over 20 MLRAs that can be trained and validated with either user’s experimental data or ARTMO internally synthesized data. Moreover, over the years, several additional modules have been added to the MLRA toolbox, such as the AL module [40], band analysis module [15], dimensionality reduction module [42]. In order to achieve an optimal LAI*_G_* estimation with a manageable training dataset, several AL techniques were applied, namely: Angle-Based Diversity [ABD], Clustering-Based Diversity [CBD], Euclidean Diversity [EBD], Entropy Query-by-Bagging [EQD], Pool Active Learning [PAL], Random Sampling [RS], Residual Active Learning [RSAL]. The description of the sample selection criteria and utility of these AL algorithms for biophysical variables retrieval were previously explained and analyzed in [40,41]. In ARTMO, the initial AL sampling configuration was set to a 10% of the input learning set of 218 samples, i.e., 22 samples. Then, for each AL method, the selected samples were added by 5 per iteration.

**Figure 1:**
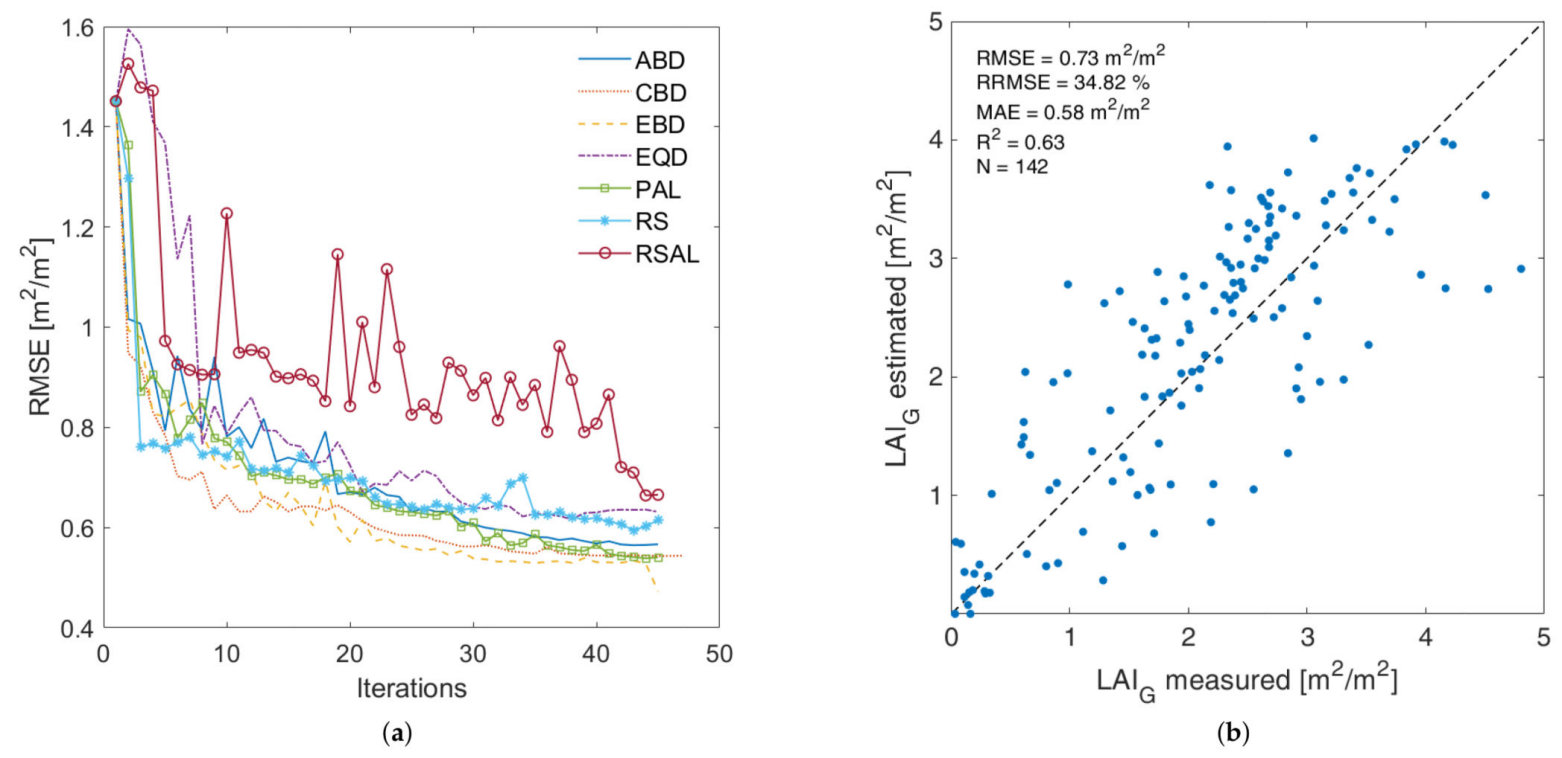
Comparison of the RMSE evolution performance of all active learning (AL) methods (**a**), and scatterplot with goodness-of-fit statistics of validation LAI*_G_* data against estimations using the best Euclidean Diversity (EBD)-reduced LAI*_G_* GPR model (**b**).

AL algorithms applied were compared in terms of accuracy and efficiency, calculating various metrics: root mean square error (RMSE), relative RMSE (RRMSE), mean absolute error (MAE), coefficient of determination (R^2^), number of samples integrating the final training set and the number of iterations run until no more improvements occur. Figure 1 (left) shows, for each AL training method, the RMSE obtained from each iteration, revealing that all processes tend to converge around 45 iterations, meaning no significant improvements were further achieved in terms of accuracy. Sharp increases in the RMSE indicate non-informative samples introduced from the input learning set that did not contribute to the model predictive capacity and therefore were discarded. In view of all AL algorithms performances, EBD was the algorithm most capable of providing a LAI*_G_* GPR model both accurate and efficient, and therefore was the one chosen to be implemented into GEE. The total number of samples needed to construct the model was 118 in addition to the initial training dataset. Thus, in total the final LAI*_G_* model was trained with 140 samples. This LAI*_G_* GPR model has also been validated against the independent validation dataset as described in [21], and summarized in Table 2. It led to an RMSE of 0.73 m^2^/m^2^ and an R^2^ of 0.63 (see Figure 1, right). It must be remarked that the model provides a somewhat poorer validation as opposed to the original LAI*_G_* model, developed with the full training dataset, reported in [21] (R^2^ of 0.70), but is still considered as valid for usage as a demonstration case into the GEE environment.

### 3.2 Gapfilling Model

In general, modeling phenological evolution represents a challenging task mainly because of time series gaps due to clouds and calibration or atmospheric correction residual errors[43–46]. Reliable gap-filling fitting functions and smoothing filters are frequently required for retrievals at the highest feasible accuracy [47,48]. Of specific interest to filling gaps in time series is the emergence of machine learning regression algorithms (MLRAs) which can serve as fitting functions. Among the multiple MLRA approaches currently available, the kernel-based methods developed in a Bayesian framework deserve special attention, such as GPR [13]. Recent studies demonstrated the effectiveness of GPR for gap-filling of biophysical parameter time series [18–20] because the hyperparameters can be optimally set for each time series (one for each pixel in the area) with a single optimization procedure. Despite its clear strategic advantage, the most important shortcomings of this technique are their (1) high computational cost and (2) memory requirements [49] for hyperparameter optimization, which grows cubically and quadratically with the number of training points, respectively [50,51]. This can become problematic in view of processing a large amount of data, such as in S2 time series tiles. Parallelization strategies for advanced computation platforms such as GEE need to be developed to speed up the GPR processing while maintaining the superior performance in terms of accuracy, being these facilities are not devised for per-pixel iterative optimization tasks.

To mitigate this computational burden and address such shortcoming and repetitive procedure, we propose the approach pursued in Belda et al. [22]. Here, the model in [28] was applied to generate LAI*_G_* time series over an agricultural region in Castile and Leon, North-West Spain, and the performance of gapfilling task using GPR per-pixel optimization (**θ***_pp_*) versus cropland-based (***θ**_pc_*) and global precalculated (***θ**_gl_*) SE-kernel hyperparameters were compared. Table 3 summarizes hyperparameters’ mean values for the different approaches, whereas Table 4 described the variation in percentage of the gapfilled time series with respect to the input ones for the different approaches. The main conclusion of this study is that using the same pre-optimized hyperparameters for all crop types, the performance degraded between 2% and 7% with respect to per-pixel optimization, whilst the processing speed is 90 times faster than the standard GPR estimations.

Since accurate spatiotemporally-explicit knowledge of vegetation phenology is critical to understand the changing trend of natural seasonal phenomena and serve for agricultural production and global change studies, this comparison becomes crucial to assess the sensitivity of the phenological parameters to the variations in the hyperparameters. The crop phenology indicators were already studied and compared for these three different approaches in [22]. Results showed analogous crop temporal patterns, with differences in start and end of growing season no more than five days. This again confirms that the reconstruction of multiple season vegetation temporal patterns are rather insensitive to fixed hyperparameters optimized over either homogeneous and heterogeneous agricultural areas. In all the evidences, the global hyperparameters for the SE kernel function can be used for the optimized implementation of the gapfilling model in Equation (10) in the GEE environment.

## 4 GEE Implementation and Assessment

GEE is an online service that applies state-of-the-art cloud computing and storage infrastructures for geospatial analysis. The archive contains a large catalog of Earth observation data which enables the scientific community to perform calculations on high numbers of images in parallel [24,52–54]. The procedure to request data, perform spatial calculations and serve the information is carried out with code developed from either JavaScript or Python APIs instructions, which are interpreted and sent by the client to Google servers as JSON request objects. The exploitation of GEE computational power is achieved by minimizing the operation on the client side and client-server data transfer, which constitutes a real bottleneck for any algorithm to be efficient. In addition, algorithms’ processing flowchart must generally be reviewed to optimize the usage of parallel computing resources with respect to client stand-alone implementation. For instance, this last step is key when migrating the client-side GPR coding to GEE, because its typical pixel-wise implementation turns out to be very inefficient. Conversely, implementing the matrix reformulation of Equations (8) and (9) proposed in Section 2.2 allows taking profit of the cloud-computing strategy for LAI estimation and LAI gap-filling over wide area at the maximum spatial resolution provided by S2 multispectral imagery. The implementation of the two models in GEE backs up entirely on the use of functionalities provided by the *ee* library, which is available in both JavaScript and Python API interfaces. This choice allows an efficient usage of the imagery catalogues GEE contains, as well as its highly paralleled computational power. Additionally, code migration between the two programming languages is almost immediate.

### 4.1 LAI_G_ Mapping

The key function of the GPR model for LAI*_G_* estimation from S2 imagery is summarized in the few code lines in Table 5. For readers interested in the methodology, the link to a toy example with the complete LAI prediction code is available in the Supplementary Material. Here, mathematical instead of standard GEE code editor notation is used for variables to ease the identification of the parameters in Equation (9). The word *var* is put between parenthesis as this variable declaration is required in Java Script but not in Python scripting.

Note that the overall number of code lines for the GPR-based prediction of the mean LAI*_G_* is incredibly low. The reason is that *ee* API provided by GEE library allows the implementation of even complicated Matrix algebra operations very easily. [TS_ID] is the *ee*.*List***()** of training samples text labels required to shape an image turned into an array back to a multispectral image format; ***XDX*** correspond to the third element of Equation (8); the input to the function, i.e., “image”, represents the element of S2 collection the model is applied to.

**Figure 2:**
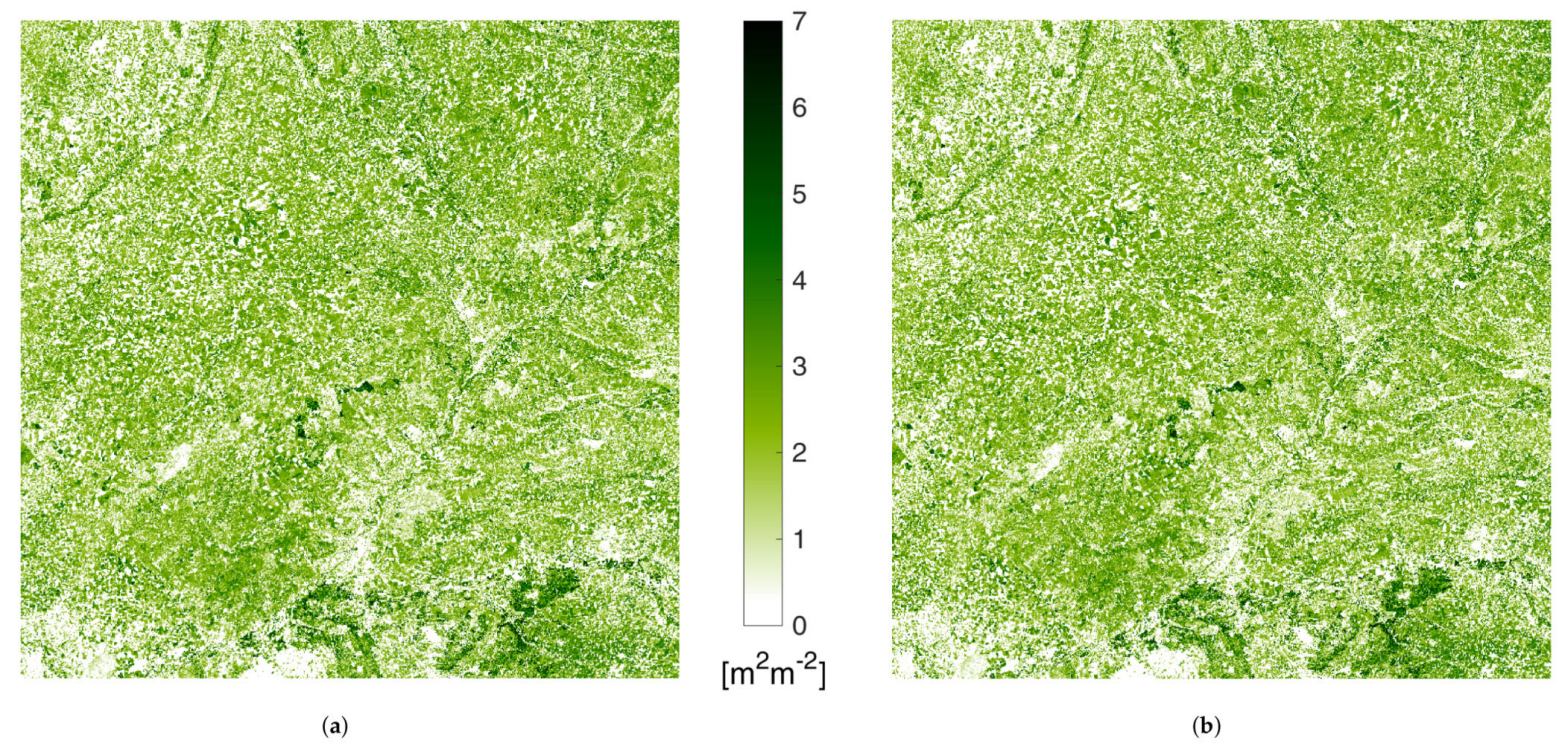
Comparison between green LAI maps retrieved by Automated Radiative Transfer Models Operator (ARTMO) (**a**) and GEE GPR coding (**b**) from S2 acquisition over tile 30TUM on 2 May 2019.

**Figure 3:**
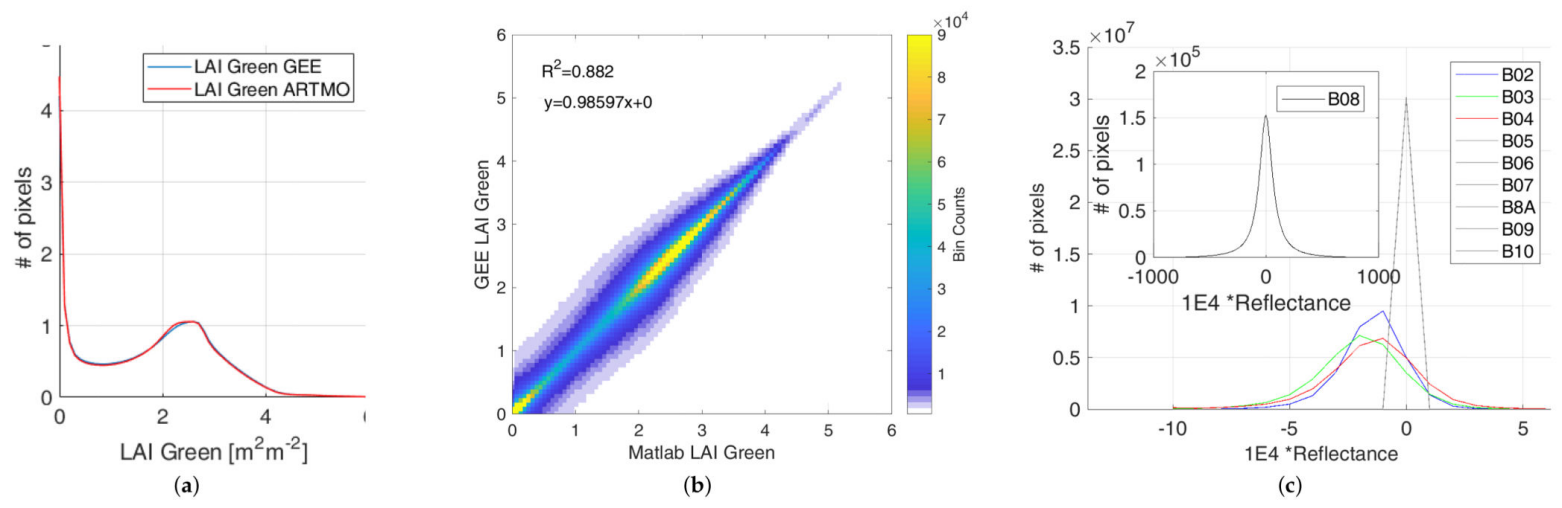
Comparison of green LAI maps estimated by ARTMO and GEE GPR implementations: histograms (**a**) and scatterplot density (**b**). In (**c**), the histogram of pixel-by-pixel difference between 10 m bands resampled at 20 m distributed by ESA and available in GEE.

A comparison of the LAI map at 20 m retrieved by ARTMO (used for the GPR model training) and by GEE implementation is given in Figure 2. The area shown corresponds to the S2 acquisition taken on 2 May 2019, over the Tile 30TUM in Castille and Leon, North Spain, corrected for the atmospheric contribution (level L2A). The input S2 imagery for ARTMO retrieval came from the S2 distribution of ESA Scientific Data Hub [55]. Input information to GEE-based estimation was the ESA L2A product directly available in the platform. The qualitative assessment from the visual inspection does not reveal any significant differences: the dynamic range of LAI*_G_* is almost identical and spatial patterns agree perfectly everywhere. The quantitative assessment of Figure 3a shows the histograms of the two LAI*_G_* maps overlap everywhere but for two narrow zones around 1 and 2.2. The analysis of the 2D histogram between the two maps of Figure 3b reveals slight differences are present, leading to a R^2^ = 0.89. The dispersion of the bin scatterplot was initially ascribed to the single-precision of GEE implementation vs. double-precision ARTMO estimations. A more careful analysis of the two input imageries revealed the existence of a slight mismatch between the 10 m bands upscaled to 20 m (B2,B3,B4,B8) distributed by ESA and those available in GEE. The histograms of the differences between the two distributions are shown in Figure 3c. Note that the discrepancies are higher for NIR(B08) than RGB bands(B04,B03,B02), being null for the 20 m native resolution bands. This difference, visually corresponding to geometric slight unmatching, generate the differences emphasized by Figure 3b. Nonetheless, the high resemblance between the two products corroborates the correctness of the GEE implementation of GPR model LAI*_G_* mean value estimation with respect to pixel’s radiance information.

The time required for ARTMO processing is in order of a few minutes, visualization in GEE takes a few seconds whereas downloading the full-resolution LAI*_G_* product might take between less than a minutes up to 2/3 min, depending on the GEE servers’ computational load in that moment. Note that for a fair comparison, the time required for L2A file downloading from Copernicus webpage [23] should be also taken into account, being this task instantaneous in GEE.

**Figure 4:**
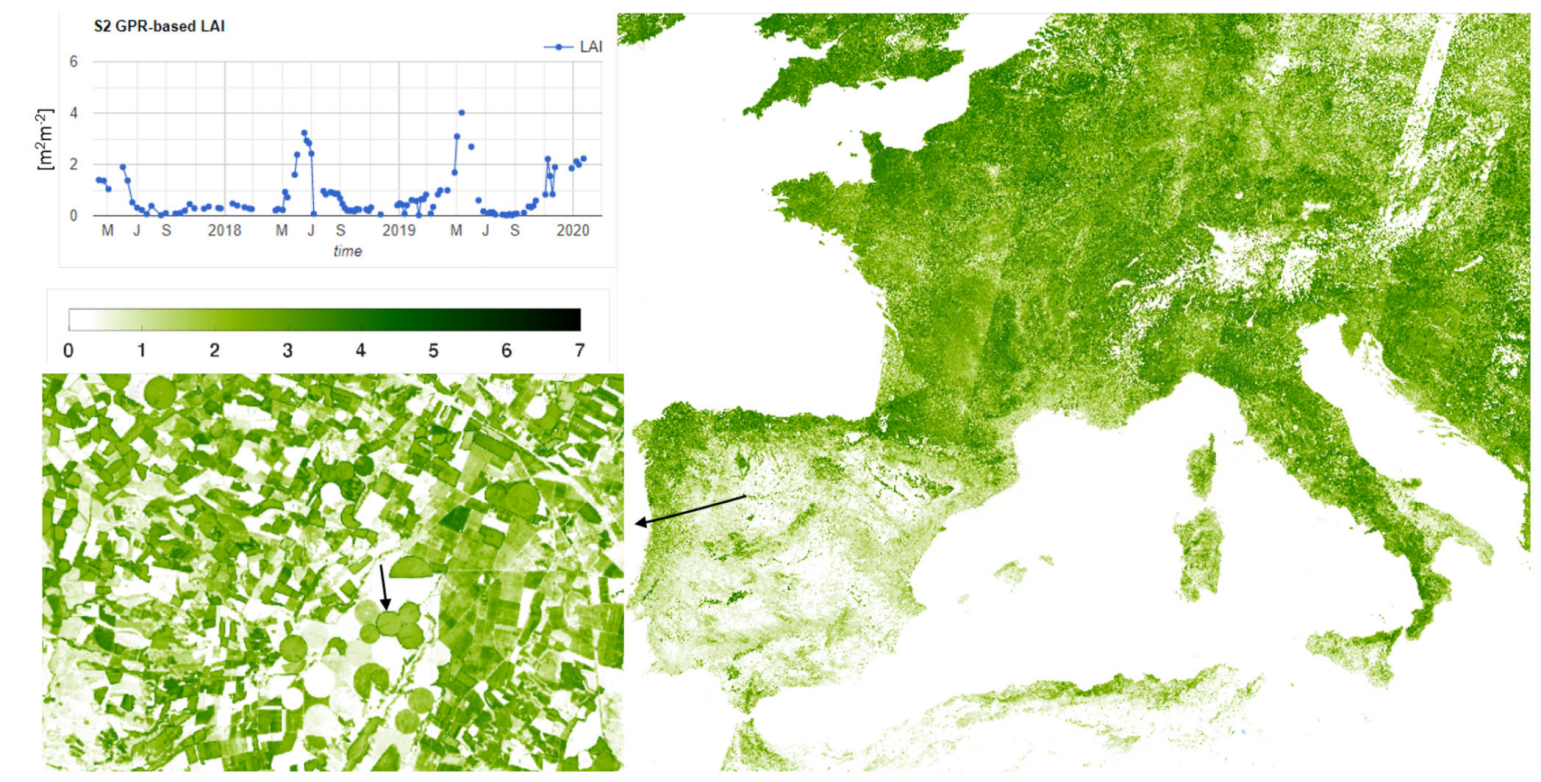
Big Scale LAI*_G_* Mosaic over Europe with 1 July to 15 July 2019 time span using maximum value strategy.

The undisputed advantage of GEE vs. any local-processing solution arises when it comes to processing very large areas. In the classical approach, this involves dealing with a very long image downloading process, computational burden, storage and mosaicking issues, pyramids generation for quick visualization. Moreover, any mistake often causes repeating this process partially or even entirely, depending on the step of the chain it takes places. Applying the LAI*_G_* model to a large collection of S2 images over a variable time span is extremely easy and the visualization of the mosaicked result available in less than one minute at the resolution active in the display panel of GEE code editor. In addition, exploring any pixel’s LAI time series at any spatial resolution becomes extremely practical. Figure 4 provides an example of the maximum LAI*_G_* value within the first two weeks of July 2019 over western Europe. The maximum resolution of the map is 20 m, as shown in the zoomed area of Castille and Leon, whereas the time series of a pixel selected within a pivot parcel highlights the possibility to identify the different crop seasons. The chance to visually compare so wide area up to so high spatial resolution in terms of a parameters obtained by an advanced machine learning modeling as GPR is something never shown before. Different climatic areas can be easily identified while moving from north to south, or from east to west, both at continental and national scales. For instance France with respect to Spain, north Spain vs. south Spain, or east vs. southwest Italy. Quite continuous maps are obtained over Mediterranean areas, which are characterized by low presence clouds in July. Yet, orbit discontinuities over more cloudy regions are visible, and along with the map gaps moving towards east Europe make such mapping approach useful but still incomplete. For this reason, after being able to implement the LAI*_G_* GPR model for S2 imagery, in the next section we tackle the problem of time series gapfilling.

### 4.2 LAI_G_ Time Series Gapfilling

It was earlier demonstrated that the SE kernel function provides high performance to fill the gaps of LAI*_G_* time series induced by cloud presence, and so can also mitigate the effects of reflectance atmospheric correction residual errors [20]. The rationale of the kernel-based approach for the time domain has been briefly described in Section 2.3, whereas the precalculated optmimum global hyperparameters *θ_gl_* to be applied for a whole S2 tile have been reported in Table 3. In the computational approach pursued in [22], each pixel is analyzed independently because the length of the time series as well as the corresponding time stamps change spatially. The main drawback of this formulation is that the dimension of covariance matrix **K** in Equation (10) is pixel-dependent. In standard sequential computation, each time series is processed separately, and any length can be nicely dealt with. In a parallel computing approach, this length variability hinders its parallel implementation in any cloud platform, in particular GEE. The reason is that in GEE any cloudy pixel is dealt with as a masked pixel (i.e., a not-a-number), and any operation involving its use generates by default a non-valid output. Should we substitute them with zeros, these new values would compromise the time series reconstruction.

**Figure 5:**
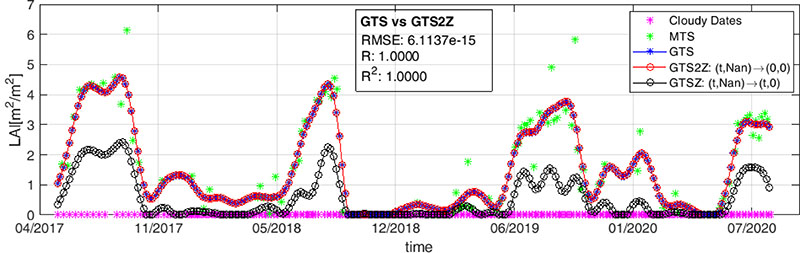
Result of GPR gapfilling of input LAI*_G_* time series—meaning values (MTS) plus cloudy acquisitions (CD)—using different approaches: standard GPR prediction (GTS), CD substitutions with 0 (GTSZ) and the proposed parallel implementation (GTS2Z).

A workaround to these two issues deals with modifying the dates of the input time series. Being the nominal time vector **t** common to all the pixels of S2 image collection over the same tile, we first convert each acquisition date in a numeric format. We choose to express it in terms of number of days with respect to the GPS time reference, i.e., 1 January 1970. As an example, according to this criterion the date 20 July 2019 becomes the number 18,097. Afterwards, we add (1) a new constant band to each S2 image of the collection corresponding to its numeric date (band t_*_), and (2) a copy of this new band where we set to 0 (i.e., not masked anymore) all the pixels labeled as cloud. Cloudy pixel identify backs up on the S2 classification product [56] distributed along with L2A multispectral reflectance (band ***t****_msk_*). Similarly, we set to zero also the reflectance values of these cloudy pixels in each element of the S2 collection. Summing up, in the time series of a generic pixels from the S2 collection, the time associated to cloudy samples is 0 as well as their multispectral radiometric values. Taking into account that the input time series for calculating the temporal Covariance matrix ***K**_t_* must not be time ordered, the contribution of all these cloudy captures to the estimation of LAI*_G_* at any S2 acquisition date, i.e., the value of *k*(0, *t_i_*) in Equation (10), becomes negligible. This is shown in Figure 5, where we analyze the LAIG time series of a pixel belonging to a vegetated area in Castille and Leon, Spain. As expected, the collection presents both meaningful (cloud-free) and non-valid (cloudy) samples. These two cases are described by the meaningful (MTS) and cloudy (CD) time series points. CD dates correspond to S2 acquisitions where the chosen pixel but not the whole tile is cloudy; completely cloudy captures over tile 30TUM have been discarded. The blue plot (GTS) describes the result obtained with the standard approach, i.e., all the cloudy samples are eliminated and only MTS is used for the GPR model prediction; in red the result provided by the proposed strategy (GTS2Z), i.e., by substituting (0,0) for each (date, LAIG) pair from a cloudy capture and performing the prediction using all the samples. The gapfilling has been performed over the whole observation period with a 5-day sampling step. It can be observed that GTS and GTS2Z plots perfectly match, and the overall difference between the two estimation is utterly negligible (RMSE = 2.9 × 10^–14^, R^2^ = 1). For the sake of completeness, we show in black (GTSZ) the result obtained when cloudy samples are set to 0 to demonstrate that this substitution leads to incorrect estimations of LAIG and should not be used.

The main advantage of the proposed workaround, i.e., GTS2Z, is that the dimension of the time series being processed for LAI*_G_* gapfilling at any time t_*_ is the same for all the pixels of the tile. This way, calculating***k**_t_* and then **α***_t_* in Equation (12) can be tackled with a parallel computing strategy.

Following the same idea described in Section 3.1, in Table 6 we provide here a few keylines of GEE pseudo-code implementing the core of the GPR-based gapfilling method. The link to a toy example with the complete gapfilling code is available in the Supplementary Material. Again, the variable names are assigned using the mathematical notation of Equations (11) and (12) to make the identification of the different terms easier: LAIG*_c_* indicates the collection of LAI*_G_* maps obtained from a S2 reflectance imagery over the same tile during a specific time interval; **1** denotes a matrix of ones; bold font defines variables which take into account all the pixels of the collection at once. The final prediction corresponds to the LAI*_G_* cloud-free map at time t_*_. Details on the GPR formulation here implemented can be found in [13]. The key steps are (1) the implementation of Equation (11) by defining the **1***_msk_* and 1_*_ vectors, and (2) the parallel calculation of *L* matrix using the Cholesky Decomposition available in *ee* library. In this case too, the matrix algebra operation can be performed by casting the multiband images from *image* to *array* data type. The final result is converted back to *image* using the *arrayProject* functions to add geocoordinates and turn it into a map. As dealing with *array* type means asking for chunks of contiguous memory [57], care must be taken to the global volume of data involved in the process. Note that the theoretical computational complexity of LAI prediction as well as gapfilling steps is *O*(*n*_3_), being *n* the number of bands for the former and time series length for the latter. For this reason, the gapfilling task over multiple-tile areas must be carried out by looping over tiles and considering one date at a time. This way, memory exceeded errors are nicely avoided.

**Figure 6:**
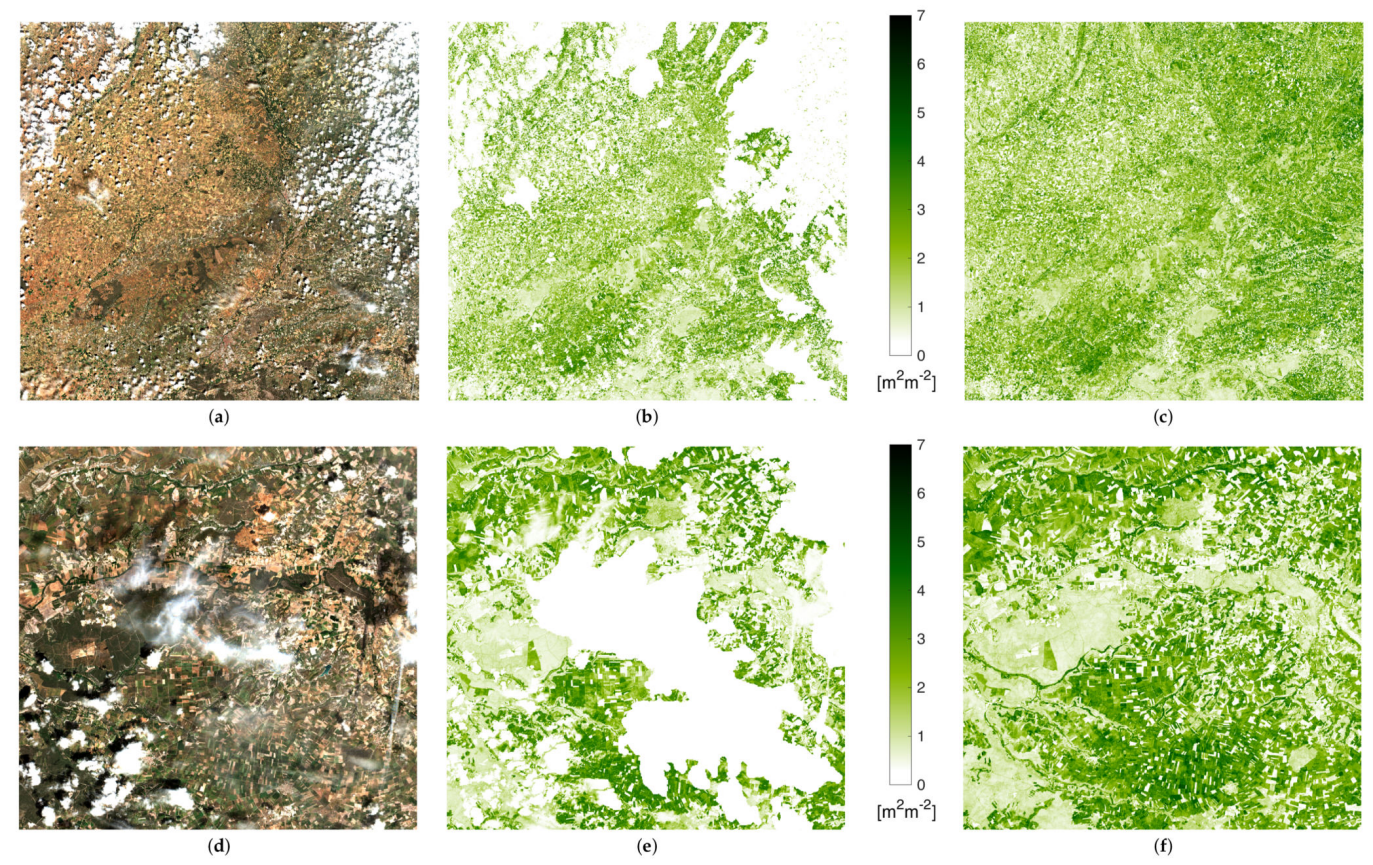
S2 acquisition over tile 30TUM on 27 May 2019: RGB (**a**), *LAI_G_* map (**b**) and gapfilled LAI*_G_* map (**c**) from GPR model applied in GEE to ± 35 dd span S2 collection. In (**d**–**f**), the same quantity zoomed onto the bottom-right corner.

**Figure 7:**
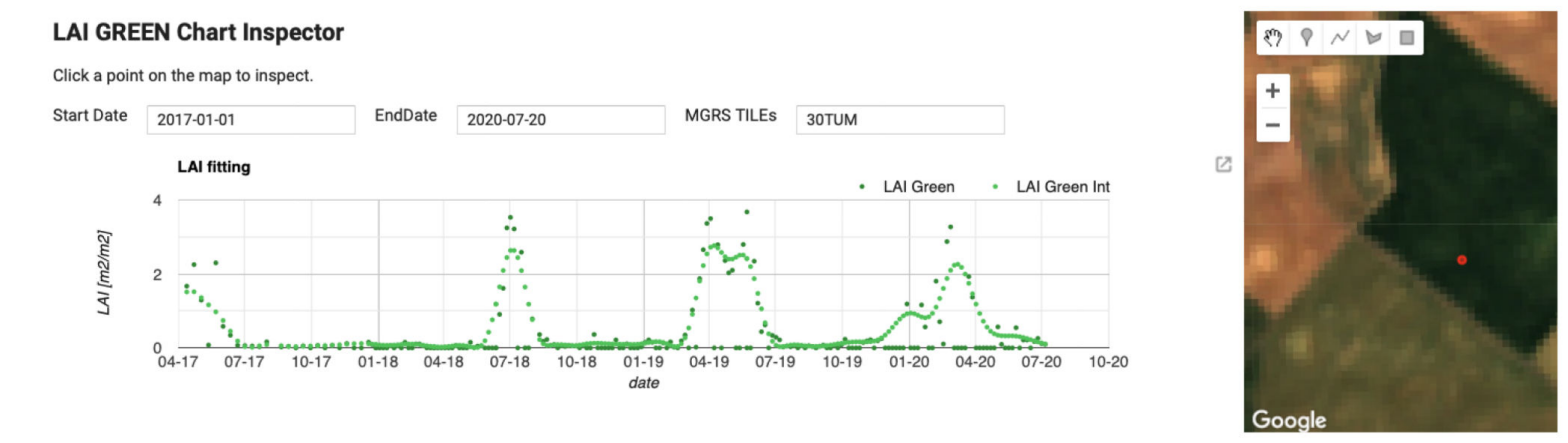
Original and gapfilled LAI*_G_* time series of a crop pixel with the area of study generated using GEE visualization APIs. The time span corresponds to the whole collection of S2 L2A imagery distributed by ESA available in GEE.

An example of the high performance of the proposed gapfilling strategy is provided in Figure 6. Here we present the case of a cloudy capture of 30TUM S2 tile over Castille and Leon taken in Spring 2019: (a) the RGB image (bands B4,B3,B2), (b) the LAI*_G_* map obtained as explained in Section 4.1, (c) the gap filled map. It can be seen as the patterns of non-cloudy areas are generally maintained, and how the new areas added perfectly match the surrounding information creating seamless mapping. Images (d), (e) and (f) detail the same information over a zoomed area of the right-bottom corner. Again, no gapfilling artifact is detectable, even over small isolated cloud often not included in the L2A classification layer. The reason is that GPR is able to identify time series isolated outliers and reduce their effect. This can be observed in the original and gapfilled time series of a pixel selected in the studied area, shown in Figure 7. The image corresponds to a screenshot of the interactive panel we developed for inspection purposes with the GEE integrated development environment (IDE). The time window corresponds to the whole collection of S2 images distributed by ESA and available in GEE, spanning from January 2017 up to July 2020, when the study here described was finished. The different crop seasons can be easily distinguished, as they correspond to clear patterns along the LAI*_G_* phenology evolution. Links to working code for LAI*_G_* estimation and gapfilling implementation are available in Supplementary Materials for interested readers.

## 5 Cloud-Free Seamless Mapping of Wide Areas

**Figure 8:**
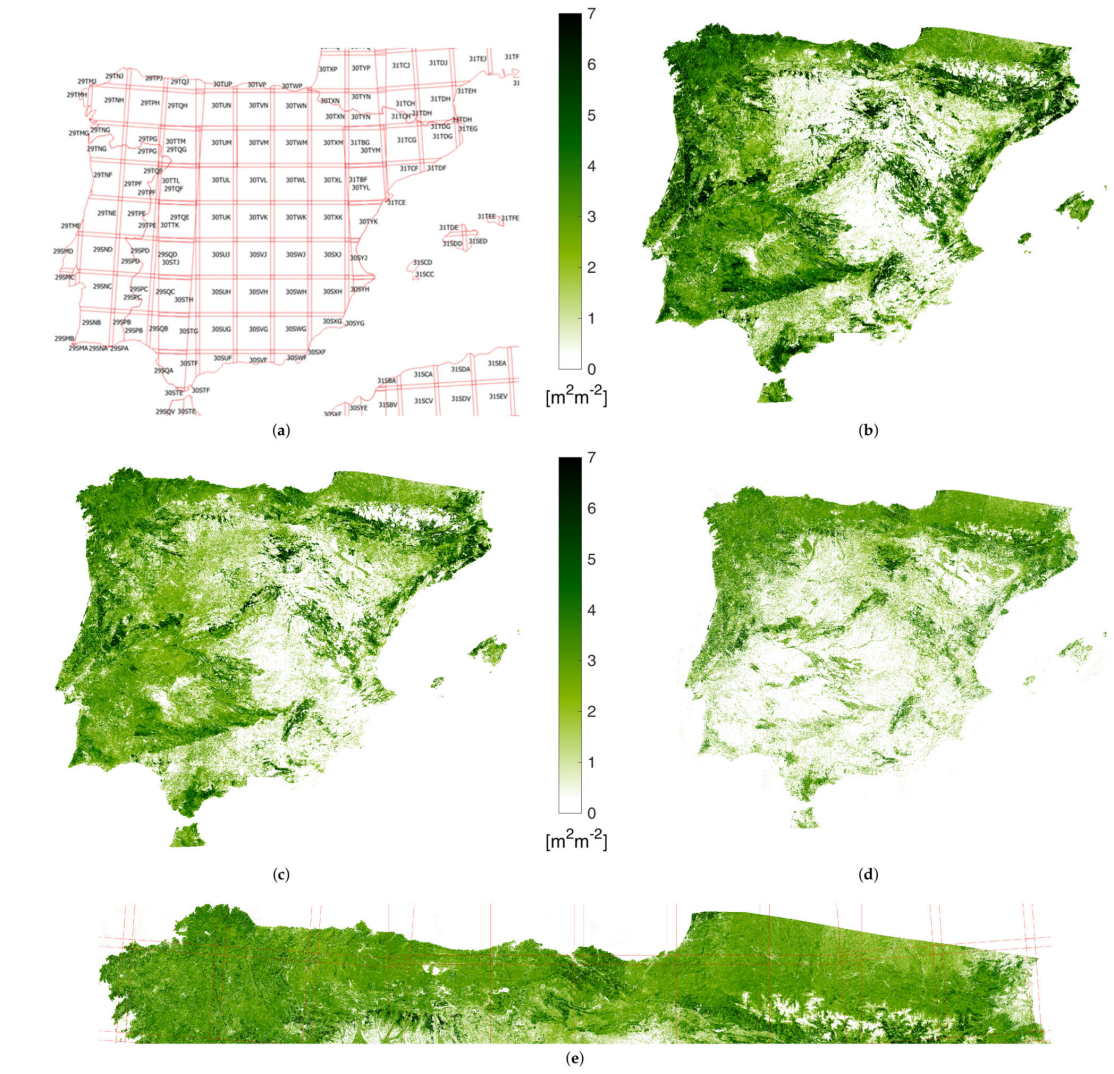
Mosaic of gapfilled green LAI maps over Iberian Peninsula from S2-tiling (**a**) on 2 February (**b**), 30 March (**c**) and 30 June (**d**), 2019. In (**e**), zoom of 30 June mosaic over the Atlantic coast and Pyrenees Area.

The aim of this section was to demonstrate the great potential of the methodology here proposed for wide areas monitoring. It is often of key importance being able to provide continuous mapping of LAI*_G_* at a specific time all over the area of interest, but the presence of clouds usually hinders or makes unfeasible the comparison of zones evolution on the same dates. The area chosen as demonstration case is the whole Iberian peninsula, then including Portugal, Spain and the south area of France along Pyrenees. In terms of extension, an overall number of 127 tiles from 6 different orbits (relative number from east to west 8, 51, 37, 80, 94, 173) are required for the complete coverage. A detail of tile distribution is shown in the top-left image (a) in Figure 8. To stress the different evolutions of LAI*_G_* according to geographical coordinates of zones, we selected 3 different dates: 2 February, 30 March and 30 June, 2019. For each date, we create a collection of S2 reflectance images within the time span given by the date ± 3 months. Note that this time span corresponds to approximately 6 times the lengthscale hyperparameter in ***θ**_gl_* (Section 3.2), which ensures all the samples that contribute meaningfully to the prediction in Equation (12) are accounted for.

The result of the LAI*_G_* mapping at 20 m for the three dates is shown in the images (b–d) of Figure 8, each one representative of a different season: Winter, Spring and Summer. The quality of the reconstructed LAI*_G_* maps is evident. No orbit artifact is detectable for the three dates, confirming the capability of the proposed methodology to guarantee a spatial continuity of the result even if it is applied in a pixel-wise fashion. Clear temporal patterns can be observed by comparing the three maps. For instance the north area of Spain and Portugal are characterized by very high values of LAI*_G_*, indicating the presence of dense forests, with respect to the central and south part of the peninsula, where seasonal vegetation growth is more common. In addition, the presence of large areas where not significant LAI*_G_* is detectable during the three seasons here monitored (and probably during autumn too) can be also observed. Note that the usage of an advanced machine learning model for LAI*_G_* such as GPR, which exploits the whole multispectral information of S2 data, allows enhancing the dynamic range of the model, overcoming saturation issues usually exhibited by models based on vegetation indexes.

Different climatic areas can also be deduced from this mapping, which resemble the distribution of the average total precipitation in the Iberian Peninsula (see Figure 69 in [58]) and confirm the identification of areas characterized by desertification in the Köppen– Geiger Climatic Classification of Iberian Peninsula (see Figure 1 in [58]). Finally, it is worth emphasizing that the spatial continuity is also maintained at the 20m resolution. The visual inspection of the maps at the maximum detail did not reveal any radiometric discontinuity. The need to visualize a wide area oblige to compress the whole information in a small representation, so that details of the maximum resolution map cannot be appreciated. This is the purpose of image (e) in Figure 8, which provide a zoom of the LAI*_G_* map of 30 June over the so-called Atlantic Mountain Range and the Pyrenees. This subset area represents a critical test site, being characterized by a severe cloud presence (probability between 60% and 70% between April and October [59]). Again, no radiometric discontinuities or artifacts can be detected both in coastal and mountainous zones, confirming the high reliability of the kernel-based gap-filling regression method we proposed over multi-tiled areas of study.

One closing comment about computational time. The processing of each tile takes about 1 min, and an average of 26 min were necessary for tile downloading to Google Drive at 20 m; the overall time required for downloading the 127 tiles composing each map was in average 26 h. When setting the output resolution to higher values, the overall time drops significantly. For instance, less than 2 h were required to get a complete LAI*_G_* map at 300 m spatial resolution.

## 6 Discussion

This work presented the integration of GPR into GEE for seamless vegetation properties mapping. The advantages of GEE as an image processing platform are unprecedented: GEE has opened a new big data paradigm for storage and analysis of open-access Earth observation (EO) data over areas with a spatial extension and at a spatial resolution that was not feasible using any desktop processing machines [24]. In the following, we will discuss its strengths and weaknesses.

In GEE, whole data collections of multiple EO missions from medium to coarse spatial resolution are available on line for free, and the user-friendly Java coding editor allows launching computational-demanding processes over multiple distributed platforms. In addition, efficient mosaicking tools make it possible to deal with raster and vector information at once for selecting specific areas to be studied, or extend the analysis to nationwide coverage [60,61]. GEE Python coding is also possible using specific wrapper of the *ee* library, giving the opportunity to link GEE data catalogue to GIS environment such as QGIS [62,63], and use it as a starting point for more advanced client-side analysis. Finally, information down- and up-loading can be carried out very efficiently using solution as Google Data Cloud or Google Drive [64]. All these aspects make GEE extremely appealing for the development of EO applications. Yet, also some inconveniences need to be addressed.

First of all, scarce supporting documentation is provided in the official webpage. Error description during debugging is not exhaustive [57], and becoming aware of library changes due to continuous updates is not straightforward. To a certain point, the bug report official channel is helpful to find how-to-do examples, but these gaps are mitigated mostly by the ever-growing numbers of users sharing their experience in forums [65] and unofficial users’ blogs [66,67]. Materials shared by the GEE community provide enlightening pieces of code that allowed us to look into *ee* methods and clarify the input-output specific formats they require. The main GEE limitation we had to circumvent is that despite the rapid progress in cloud-based algorithm development, not all MLRAs have been developed into the GEE ecosystem. Particularly, the GPR algorithm appeared to be lacking, which eventually became the rationale of investing into its implementation and the here presented work.

Secondly, although GPR is a highly competitive regression algorithm and has appealing advantages over other MLRAs such as uncertainty estimates and band ranking properties [3,8,14–16,26], one reason why GPR did not find its way yet into GEE is likely due to the heavy usage of contiguous memory allocation required by the GEE *array* data type. Matrix algebra operations involved in the GPR regression implementation cannot be performed using directly the *image* data type in which all the GEE catalog imageries are made available. To apply these specific algebra operations, the conversion from *image* to *array* data type is compulsory. Unfortunately, due to the particular usage of computational memory *array* type performs, using *array* type generates “memory exceeded” error messages much more frequently than using *image* type, even if the number of acquisitions to be processed is low (<40). To avoid these errors, computational workarounds must be devised.

In order to bypass these memory-related limitations, we had to introduce multiple adaptations, which are summarized as follows. We (1) expanded the formulation of standard GPR, (2) aggregated all terms independent of pixel’s hyperspectral information that can be precalculated to avoid repeating cumbersome operations for each pixel, (3) performed data manipulation that can be carried out using *image* datatype format before moving to *array* data type, (4) implemented GPR into a matrix algebra formulation and (5) converted the results back to *image* format adding coordinates information, mandatory for mapping purposes. These main steps have been followed for the implementation of the LAI*_G_* model, but also for the gapfilling technique based on GPR, and constitute the result of pursuing a non-straightforward optimization of GEE coding. This is the main reason why we decided to add a few lines of pseudo-code (Tables 5 and 6), where we used mathematical symbology of Section 2 for variable names and amethyst colored for specific *ee* library functions. The scarce documentation and the few examples available online on this subject made complex algorithm development challenging, and sharing the core part is going to be key for anyone interested in implementing its own GPR model.

On the bright side, the presented workflow provides a generic strategy for importing any SE kernel-based GPR model in GEE. To demonstrate the functioning of the workflow, here we focused on the implementation of the LAI*_G_* model for national mapping. This demonstration case was chosen for two reasons. Firstly, LAI*_G_* is a key vegetation variable for many applications dealing with permanent (e.g., forests) and seasonal (e.g., croplands) vegetation phenology evolution. Secondly, the imported LAI*_G_* model is trustworthy for wide area mapping as: (1) it was trained on agriculture areas, bare soils and dense forests, allowing to cover a dynamic range of this parameter from 0 up to 10 [m^2^m^–2^] [21], and (2) a thorough assessment on the hyperparameter optimization was conducted and reported in [20,22]. Although the LAI*_G_* product was shown to be consistent, in principle any kind of vegetation property can be mapped as long as the model is accurate and robust [10]. In this respect and moving ahead towards mapping of other biophysical variables, once having an already trained GPR model at disposal, it is possible to integrate it into the developed workflow after substituting the new hyperparameter values and the corresponding training samples and normalization matrices. Although the latter step still requires manual implementation, it is foreseen that in the near future these steps will be further optimized and automated so that eventually GPR models can be smoothly imported into GEE. One last warning deals with the maximum number of samples the GPR model can contain. Keeping it below 150 prevents out-of-memory errors from occurring frequently. As demonstrated in Section 3.1, active learning optimization constitutes an efficient tool to slim down heavy models but preserve the original training set diversity. Concerning the estimation of GPR uncertainty described in Equation (2), which represents a property only GPRs offer with respect to any other ML technique, a deeper development is still needed. First tests allowed its estimation for LAI model only over small areas, but the higher amount of information data to be kept in memory makes this issue still pending for multi-tile mapping.

To this end, an alternative research line to be explored in the future deals with using Bayesian Neural Networks, which are currently the state-of-the-art for estimating NN predictive uncertainty [68,69]. Recent works have shown the tight relationship between NNs and GPs [70] and how in particular scenarios NNs can outperform GPs [71]. The limited number of samples in case only in-situ data are to be used might still represent an issue for proper training purposes, but hybrid solutions able to blend simulated information and real data might offer a feasible workaround.

Another point worth addressing is the resolved temporal aspect. The proposed gapfilling strategy yielded promising and consistent results. The examples of seamless mosaicking of cloud-free LAI*_G_* collections demonstrate the great potentials of this regression technique as a gap filler. Although this approach was so far only demonstrated over small areas within the same S2 tile [20,22], this work presents the first proof that it is robust to radiometric spatial discontinuities over very large areas even if applied pixel-wise, and hence suitable to blend information from different S2 orbits and perform high-quality large-scale mapping. Despite its promising perspectives, it must also be remarked that the gapfilling processing chain can still be improved. For instance, residual atmospheric errors affecting input reflectance may generate inconsistent intervals along the time series. The GPR model assumes all of them are valid samples, and performs a smoothing effect that may lead to underestimate local LAI*_G_* peaks. This can be observed by comparing original and gapfilled LAI*_G_* time series shown in Figure 7. As an attempt to minimize the effect of these unlikely outliers, an additional cleaning step before the gapfilling procedure, or even an iterative or two-step gapfilling approach might be considered.

A final aspect that merits further consideration is the all-comprehensive generic model we trained over multiple vegetated land covers [28] to ensure consistent any-image processing. As an alternative strategy, rather than relying on one generic model, the use multiple light models might also be considered. For instance, coarse scale classification map from MODIS or Corine can segment the area of interest to enable running multiple GPR models, each of them specialized for the LAI calculation over a specific vegetation class. This approach would not only help to achieve a more reliable information using lighter, dedicated models with beneficial computational time. Moving towards land cover specific processing also opens the possibility to develop further processing schemes depending on the land cover type. For instance for croplands one can think of the determination of key phenological descriptors such as start-of-season, end-of-season or area under the curve [72–76]; and for evergreen forests the detection of disturbances (e.g., logging, fires) [77–81] or sudden discontinuities [82]. There is no doubt that it will eventually become possible to combine all these advanced processing schemes into the GEE framework. Moving further along this line, the usage of lighter GPR models can pave the way towards a multi-model framework to retrieve and combine multiple vegetation variables over the same area. This is of interest for a range of purposes. For instance, apart from LAI*_G_*, other vegetation variables such as chlorophyll, fractional vegetation cover or fAPAR are considered of key importance for monitoring applications, e.g., related to crop productivity and safeguarding food security, for the estimation of the gross primary production (GPP) at ecosystem level [83], and ultimately for the estimation of carbon sequestration at global scale [84,85].

## 7 Conclusions

This paper presents a workflow for the implementation of GPR models into the GEE cloud platform. While GPR has emerged as a powerful machine learning regression algorithm for processing optical satellite data into biophysical variables, it is not yet part of the GEE ecosystem. To make this integration possible, it has been necessary to: (1) review the standard GPR regression formulation to achieve a factorization suitable for a parallel computing, and (2) implement the corresponding matrix algebra transformation using *ee* library APIs optimized for server-side distributed operations. As a showcase, we subsequently integrated an earlier-developed GPR model for the mapping of LAI*_G_* derived from 20 m Sentinel-2 data. We used active learning techniques to reduce the original training set without losing diversity, and hence achieved a light model fulfilling GEE memory restriction. With the developed workflow, not only on-the-fly any region across the world can be mapped at a resolution of 20 m, but by including temporal processing also gap-filled, i.e., without the occurrence of clouds. Thanks to the computational power of GEE and the fitting efficiency of the GPR model, perspectives are opened that in the near future any locally trained GPR model can be plugged into GEE for the spatiotemporal mapping of any retrievable biophysical variable.

## Supplementary Material

For readers interested in the methodology, a toy example of the JavaScript code of the full processing chain for LAI cloud-free map generation is available online at https://code.earthengine.google.com/7bc6df888eaa89e8312338bf9e8069fa, and for gapfilled time series generation at: https://code.earthengine.google.com/0473e69965806420c940cec206494236.

## Figures and Tables

**Table 1:** Overview of field campaigns for leaf area index (LAI*_G_*) collection used for training the Gaussian process regression (GPR) retrieval models.

Location	Period	#Points	Range	Instrument	Vegetation Type	Spectral Data
Barrax, Spain	3 July	102	0.4–6.2	LAI-2000	Alfalfa, corn, garlic, onion, potato, sugar beet, wheat	HyMap
Valencia, Spain	May–17 November	34	0.41–5.41	LAI-2200	Alfalfa, artichoke, lettuce, onion, potato	S2
Biely $\text{Kr}í\tilde{\text{z}}$, Czech Republic	16 August	7	5.3–9.3	LAI-2200	Spruce forest	S2
Foggia, Italy	17 March	6	3.08–4.23	LAI-2200	Wheat	S2
Poznań, Poland	17 July	6	2.69–4.2	LAI-2200	Maize, triticale, wheat	S2
Kiev Oblast, Ukraine	18 June	3	0.27–0.56	DHP	Maize, soybean	S2
Toulouse, France	18 August	1	1.77	DHP	Maize	S2

**Table 2:** Overview of campaigns for LAI*_G_* in-situ data collection used for LAI*_G_* GPR model validation.

Location	Period	#Points	Range	Instrument	Vegetation Type	Spectral Data
Toulouse, France	Nov17/Mar-May-Jul-Aug 18	52	0.03–3.84	DHP	Maize, soybean, sunflower	S2
Poznań, Poland	Apr-Jun-Aug 18	50	0.96–4.23	LAI-2200	Beetroot, maize, triticale, wheat	S2
Kiev Oblast, Ukraine	May-Jun-Aug 18	40	0.04–4.81	DHP	Maize, soybean, sunflower, wheat	S2

**Table 3:** Averaged hyperparameters estimated using fixed crop-type and global approaches : *σ_t_* defines the gap-filled time series smoothness, *σ_st_* is the amplitude scaling factor and *σ_n_* accounts for the noise variance

	Wheat	Corn	Barley	Sunflower	Rape	Pea	Alfalfa	Beet	Potato	Global
*σ_t_*	32.6018	41.0726	36.0351	23.0815	35.0548	23.9367	29.8602	47.3544	25.5081	32.7282
*σ_st_*	0.8776	1.0018	0.8395	0.5670	1.2058	0.8415	0.6465	1.1465	1.1870	0.9237
*σ_n_*	0.3377	0.4395	0.2833	0.2355	0.5085	0.2778	0.4028	0.3794	0.3620	0.3585

**Table 4:** Variation in percentage of LAI_G_ obtained with precalculated hyperparameters with respect to the original LAI time series (lowest values in bold). Last column exhibits the variance in the percentage.

Crop Type	Per-pixel Hyperpar.	Averaged Hyperparameters										Variance
		Wheat	Corn	Barley	Sunflower	Rape	Pea	Alfalfa	Beet	Potato	Global	
**Wheat**	**9.064**	10.078	10.845	10.240	9.072	10.372	8.851	10.519	10.996	8.995	10.097	0.787
**Corn**	**10.660**	10.794	11.614	10.952	9.821	11.100	9.675	11.174	11.752	9.812	10.813	0.708
**Barley**	**8.206**	8.593	9.282	8.707	7.932	8.834	7.739	9.058	9.435	7.826	8.608	0.580
**Sunflower**	**8.455**	11.238	12.366	11.474	9.928	11.642	9.752	11.646	12.633	9.929	11.263	1.265
**Rape**	**10.222**	10.798	11.483	10.950	9.576	11.070	9.351	11.207	11.598	9.573	10.816	0.799
**Pea**	**7.634**	10.017	11.601	10.314	8.719	10.557	8.485	10.671	12.026	8.624	10.050	1.371
**Alfalfa**	**11.833**	14.001	14.999	14.222	12.659	14.368	12.496	14.360	15.210	12.734	14.024	1.108
**Beet**	**8.975**	8.975	9.629	9.083	8.207	9.223	8.054	9.389	9.714	8.149	8.991	0.577
**Potato**	**7.477**	9.456	10.566	9.647	8.311	9.848	8.130	9.993	10.769	8.262	9.481	1.070

**Table 5:** Lines of pseudo-code of GPR core implementation in Google Earth Engine (GEE) with mathematical notation for variable easy identification.

(**var**) calculate_LAI_GREEN = function(image){
(**var**) $X_{*}^{T}D$ =image.multiply(**D**).toArray().toArray(1);
(**var**) X* = image.toArray().toArray(1);
(**var**) Term1 = $X_{*}^{T}D$.matrixTranspose().matrixMultiply(X*).arrayProject([0]).multiply(—0.5).exp().multiply($\sigma_{f}^{2}$)
(**var**) PtTDX= ee.Image(**X**).matrixMultiply($X_{*}^{T}D$).arrayProject([0]).arrayFlatten([TS_ID]);
(**var**) K* = PtTDX.subtract(**XDX**.multiply(0.5)).exp().toArray()
(**var**) f(X*) = K*.arrayDotProduct(**α**.toArray()).multiply(Term1).toArray(1).arrayProject([0]).arrayFlatten([[’LAIG’]]);
**return** image.select(‘LAIG’)}

**Table 6:** Lines of pseudo-code of GPR gap-filling core implementation in GEE with mathematical notation for variable easy identification.

(**var**) N_*t*_=LAIG_*c*_.size();
(**var**) **1**_*msk*_ = *t*_*msk*_.multiply(0).add(1.0);
(**var**) 1* = t_*_ multiply(0).add(1.0);
(**var**) **I** = ee.Image(ee.Array.identity(N_*t*_));
(**var**) prod = *t_msk_*.matrixMultiply(**1**_*msk*_.matrixTranspose());
(**var**) **K**_*t*_ = *prod*.subtract(prod.matrixTranspose()).pow(2).multiply(*D*_t_).multiply(–0.5).exp().multiply(*σ_f t_*);
(**var**) **L** = **I**.multiply($\sigma_{nt}^{2}$).add(**K**_*t*_).matrixCholeskyDecomposition();
(**var**) **α**_*tmp*_ = **L**.matrixInverse().matrixMultiply(*LAIG*_*c*_.toBands().unmask().toArray().toArray(1));
(**var**) **α**_*t*_ = **L**.matrixTranspose().matrixInverse().matrixMultiply(**α**_*tmp*_);
(**var**) T* = t_*_.matrixMultiply(**1**_*msk*_.matrixTranspose());
(**var**) **T**_*msk*_ = *t*_*msk*_.matrixMultiply(**1**_._matrixTranspose()).matrixTranspose();
(**var**) K*= T*.subtract(**T**_*msk*_).pow(2).multiply(*D*_*t*_).multiply(–0.5).exp().multiply(*σ*_*f t*_);
(**var**) *LAIG*(t_*_) = K*.matrixMultiply(**α**_*t*_).arrayProject([0]).arrayFlatten([[’LAIG’]]);
